# Establishment of hepatocellular carcinoma patient-derived xenografts from image-guided percutaneous biopsies

**DOI:** 10.1038/s41598-019-47104-9

**Published:** 2019-07-22

**Authors:** David J. Tischfield, Daniel Ackerman, Michael Noji, James X. Chen, Omar Johnson, Nicholas R. Perkons, Gregory J. Nadolski, Stephen J. Hunt, Michael C. Soulen, Emma E. Furth, Terence P. Gade

**Affiliations:** 1 Penn Image-Guided Interventions Laboratory, Perelman School of Medicine at the University of Pennsylvania, 3400 Spruce St., Philadelphia, PA 19104 USA; 2 Department of Radiology, Perelman School of Medicine at the University of Pennsylvania, 3400 Spruce St., Philadelphia, PA 19104 USA; 3 Department of Bioengineering, 210S 33rd St., Suite 240 Skirkanich Hall, Philadelphia, PA 19104 USA; 4 Department of Pathology and Laboratory Medicine, Perelman School of Medicine at the University of Pennsylvania, 3400 Spruce St., Philadelphia, PA 19104 USA

**Keywords:** Hepatocellular carcinoma, Cancer models

## Abstract

While patient-derived xenograft (PDX) models of hepatocellular carcinoma (HCC) have been successfully generated from resected tissues, no reliable methods have been reported for the generation of PDXs from patients who are not candidates for resection and represent the vast majority of patients with HCC. Here we compare two methods for the creation of PDXs from HCC biopsies and find that implantation of whole biopsy samples without the addition of basement membrane matrix favors the formation of PDX tumors that resemble Epstein-Barr virus (EBV)-driven B-cell lymphomas rather than HCC tumors. In contrast, implantation with Matrigel supports growth of HCC cells and leads to a high rate of HCC tumor formation from these biopsies. We validate the resulting PDXs, confirm their fidelity to the patients’ disease and conclude that minimally invasive percutaneous liver biopsies can be used with relatively high efficiency to generate PDXs of HCC.

## Introduction

The high failure rate of clinical trials is a costly feature of cancer drug development and can be largely attributed to limited drug efficacy in patients^1^. Given the large treatment effects typically seen in pre-clinical studies, this failure rate points to significant shortcomings in currently available preclinical models^2^. Commonly used panels of cancer cell lines and *in vivo* models, including cell line xenografts and genetically engineered mouse models, fail to capture the extraordinary diversity observed in different types of cancer, which can be classified into hundreds of subtypes and an even greater number of distinct genetic entities. In addition, cancer cell lines often evolve substantially through hundreds of generations of *in vitro* culture^3^. Effects seen in these models may be difficult to reproduce in patient populations which are known to harbor a far more heterogeneous disease^4^. The development of patient derived xenografts (PDXs), which involves implanting human cancer samples into immunocompromised mice, addresses some of these deficiencies by avoiding the selective pressure of *in vitro* culture and by allowing the development of large PDX cohorts matching the relevant patient population^2,5^. PDXs may be employed to predict the clinical response of individual patients (“precision medicine”) or to perform randomized trials in mice to more accurately define response to therapy across a broad range of genetically distinct cancers.

Hepatocellular carcinoma (HCC) is a highly heterogeneous disease with an extensive diversity of somatic mutations. HCC PDXs have been employed to identify HCC tumor initiating cell populations^6^, to develop methods for the detection of cancer gene amplifications^7^, to test the antitumor activity of novel inhibitors^8^ as well as to evaluate the effects of sorafenib and regorafenib, two FDA approved treatments for advanced stage HCC^9^. However, while most HCC patients in the United States present with intermediate or advanced disease and are not candidates for resection, a large majority of PDX models of HCC are generated from resected tumors, as this allows for the collection of sufficient tissue for implantation. Because surgical resection is a curative treatment option available to fewer than 20% of patients, selecting this population for model generation may introduce bias into subsequent studies. Although generating PDX models from biopsy samples is technically challenging due to the small quantities of tumor tissue obtained, doing so would allow pre-clinical research to be performed on tumors more representative of the patient population most in need of better treatment options.

The creation of murine PDX models requires rigorous validation of resulting tumors to confirm their fidelity to the cancer of interest. In particular, human samples from a variety of cancer types were found to yield Epstein-Barr virus (EBV) associated lymphomas when implanted into NOD *scid* gamma (NSG) mice. These lymphomas are known to occur after implantation of HCC samples^10^ and thought to originate from intra-tumoral B lymphocytes that have reactivated a latent EBV infection. This phenomenon shares similarities to human B-cell lymphomas in immunocompromised patients, in which EBV-transformed B-cells escape apoptosis and grow opportunistically in the absence of T-cell surveillance^11–13^. The susceptibility of immunocompromised mice to engraftment of EBV-transformed cells, the high rate of EBV infection in humans (>90%) and the presence of B lymphocytes in HCC samples^10^ necessitate the development of appropriate methodologies that identify the formation of lymphoid PDXs.

We endeavored to derive PDX models from biopsies of intermediate and advanced stage HCC (Barcelona Clinic Liver Cancer Staging System B and C) patients who are not candidates for resection. We implanted patient biopsy samples using two different techniques and characterized the resulting tumors using *in situ* hybridization (ISH) against EBV RNA, CD45 and CD3/CD20 as well as several established HCC markers. Our primary objective was to demonstrate that PDXs can be derived from percutaneous biopsies of HCC and that methodological improvements can increase the rate of success.

## Results

**Figure 1:**
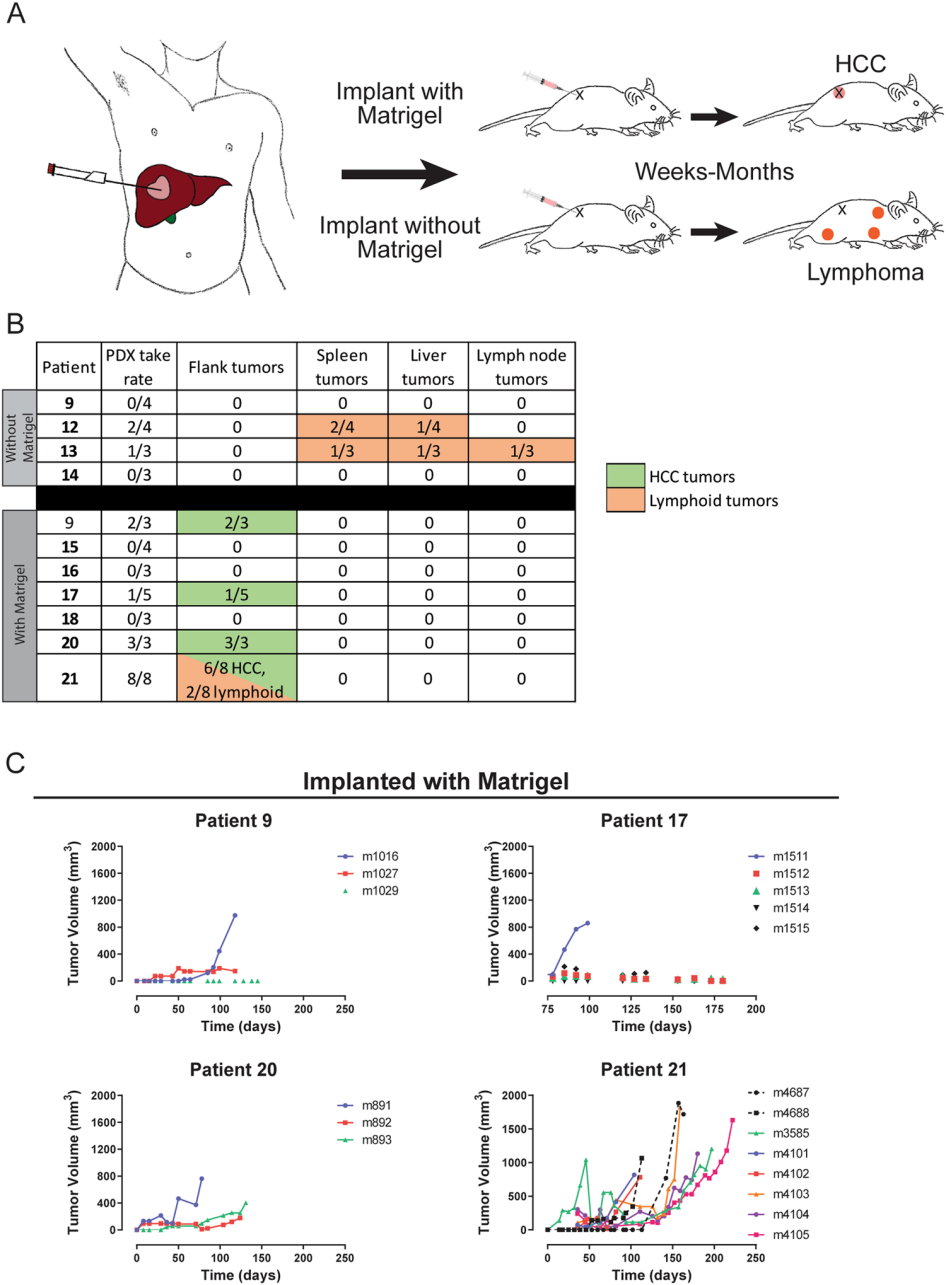
Illustration of study design and outcomes of biopsy engraftment. (**A**) Illustration of biopsy procedure and implantation process. As part of an observational clinical trial, tumor biopsies were obtained from patients with HCC immediately prior to transarterial chemoembolization using an 18 gauge BioPince core biopsy needle. Individual biopsies were then mixed with Matrigel and injected subcutaneously into the flanks of NSG mice. Early attempts at injecting biopsy samples without Matrigel resulted in the growth of lymphoid tumors at sites distant from the injection site. Upon the addition of Matrigel, we observed growth of tumors at the site of injection. (**B**) Table of the tumor take rate as well as the number tumors identified in each location for NSG mice implanted using two different protocols. Biopsies were implanted with or without Matrigel from a total 11 different patients. Dark orange or dark green indicates tumor lymphoid or HCC/CHC tumor development, respectively. (**C**) Growth curves for subcutaneous flank tumors that developed after biopsy implantation using Matrigel. Connected plot points identify tumors that eventually grew to sizes above 200 mm3. Solid lines indicate growth of tumors that were subsequently characterized as HCCs while dotted lines indicate growth of tumors that were subsequently characterized as lymphoid.

**Figure 2:**
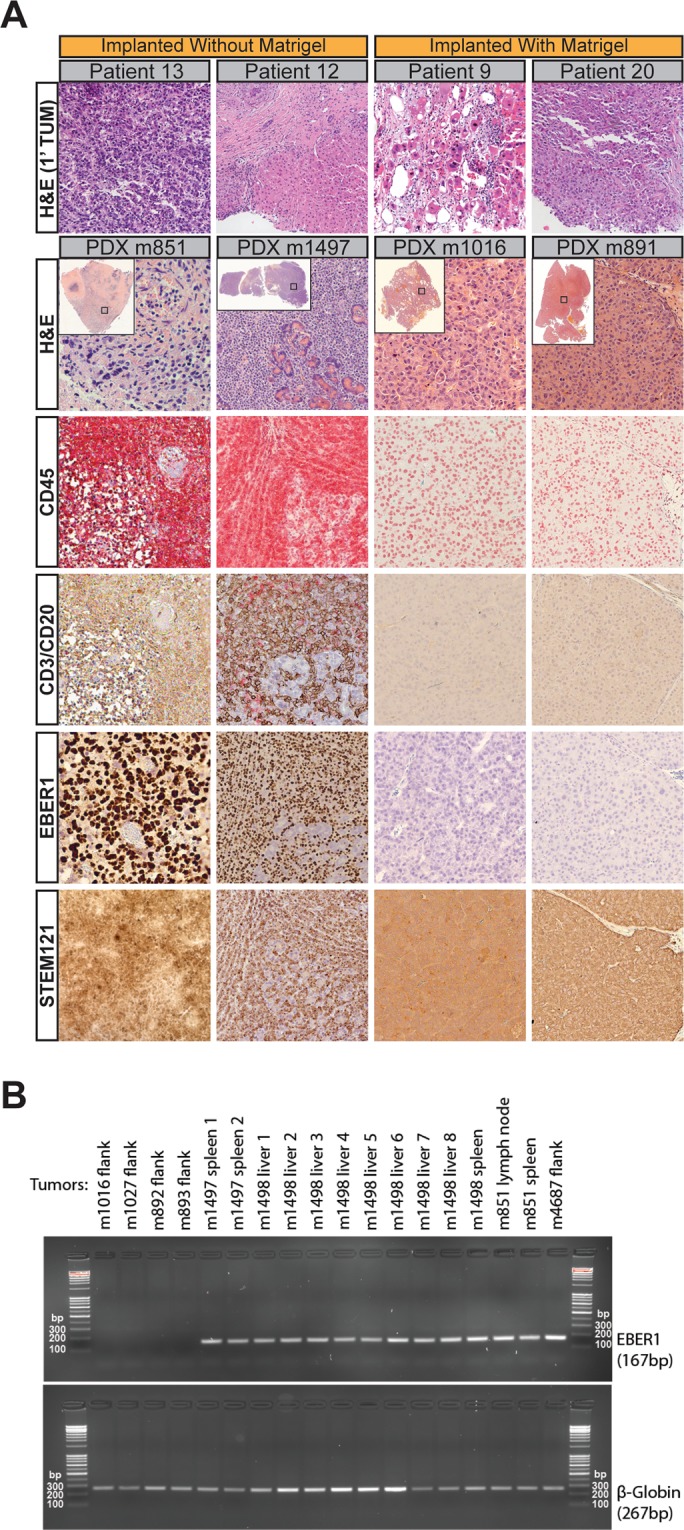
Histopathological characterization of patient-derived xenografts generated with and without Matrigel. (**A**) Representative H&E sections (x200) of HCC/CHC tumor biopsies from four different patients (top row) and the corresponding xenografts (second row) that resulted when implanted with Matrigel (right two columns) and without Matrigel (left two columns). PDXs: m851 (lymph node metastasis), m1497 (splenic metastasis), m1016 (flank tumor) and m891 (flank tumor). Whereas the xenografts generated with Matrigel morphologically resembled the matching patient biopsies, xenografts generated without Matrigel resembled lymphoid neoplasms. Montages of each xenograft specimen are shown in the top left corner of each xenografts’ H&E together with a small black box indicating the region from which the representative images were taken. Rows three and four show immunohistochemical staining for CD45 (red; general leukocyte marker) and co-staining for human CD3 (red; T-lymphocytes) as well as human CD20 (brown; B-lymphocytes), respectively. Row five shows *in-situ* hybridization for EBER1, an Epstein-Barr virus-encoded small RNA. Row 6 shows immunohistochemical staining of STEM121, a marker of human cell engraftment that can be used to differentiate mouse from human cells. While all four xenografts appropriately express STEM121, only xenografts generated without Matrigel express leukocyte markers. (**B**) PCR for EBER1 in PDX-derived tumors demonstrates positivity for tumors demonstrating histologic features of a lymphoid neoplasm but not for tumors demonstrating histologic features of HCC.

Herein, we report data derived from the first 11 patients with imaging diagnoses of HCC in our Institutional Review Board (IRB)-approved observational clinical trial. From the first four subjects, a total of 14 biopsy cores were implanted into the right flanks of 14 male NSG mice. Following implantation, tumor growth was monitored weekly through caliper measurements. Of those, the biopsies implanted from 2 patients led to tumor formation in 3 mice (Fig. 1). These mice (m1497, m1498 and m851) formed tumors in the lymph nodes, spleen and liver, which were identified on necropsy (Supplementary Table 1). Only a single mouse grew a palpable tumor near the site of implantation that sustained a size greater than 200 mm3 (Figs 1B and 2A). On necropsy, this tumor (m851, Patient 13) was determined to be an axillary lymph node metastasis. Examination of the resected PDX tissues revealed monomorphic populations of small mononuclear cells with nuclear atypia, which differed significantly in appearance from their matched HCC biopsies and were consistent with lymphoid neoplasms (Fig. 2A).

Given these findings, 29 biopsy cores from the next seven subjects were implanted as a combination of the core biopsy and 50% Matrigel. Of these, the biopsies implanted from 4 patients led to tumor formation in 14 mice (Fig. 1B,C, Supplementary Table 1). In contrast to implants formed without Matrigel, each of the tumors developed subcutaneously at the site of implantation (right flank, Fig. 1B,C, Supplementary Table 1). Twelve of these tumors strongly resembled the matching patient biopsy, and displayed histologic features consistent with HCC including wide trabeculae, prominent pseudoglandular or acinar growth patterns, cytologic atypia, and loss of the reticulin network (Fig. 2, Supplementary Table 1). Of the remaining two tumors generated through implantation with Matrigel, both displayed features consistent with lymphoid neoplasms (Supplementary Table 1).

In light of previous studies showing that PDXs in immunodeficient mice are vulnerable to EBV-driven B-cell lymphomas arising from the parent tissue of origin^10,14^, we evaluated the expression of leukocyte markers in our xenograft tissues by immunohistochemistry (IHC, Supplementary Table 1). Xenografts arising from biopsies implanted without Matrigel uniformly expressed CD45, whereas twelve out of the fourteen xenografts implanted with Matrigel did not (Fig. 2A, Supplementary Table 1). Further characterization with human-CD3/CD20 co-staining showed that CD45^+^ tumors were composed predominantly of CD20+ cells with a minority of cells expressing CD3, consistent with B-cell lymphomas (Fig. 2A). We also evaluated our xenografts for evidence of EBV infection by ISH or polymerase chain reaction (PCR) for EBV-encoded RNA (EBER1) and found that the human-derived lymphoid tumors were EBER1 positive; however, no signal could be detected in the CD45^−^ xenografts (Fig. 2A,B). These results suggest that xenotransplantation of percutaneous HCC biopsies into immunodeficient mice is vulnerable to the development of human B-cell lymphomas due to reactivation of latent EBV in intratumoral passenger B-lymphocytes, and that the addition of Matrigel helps to mitigate this phenomenon.

**Figure 3:**
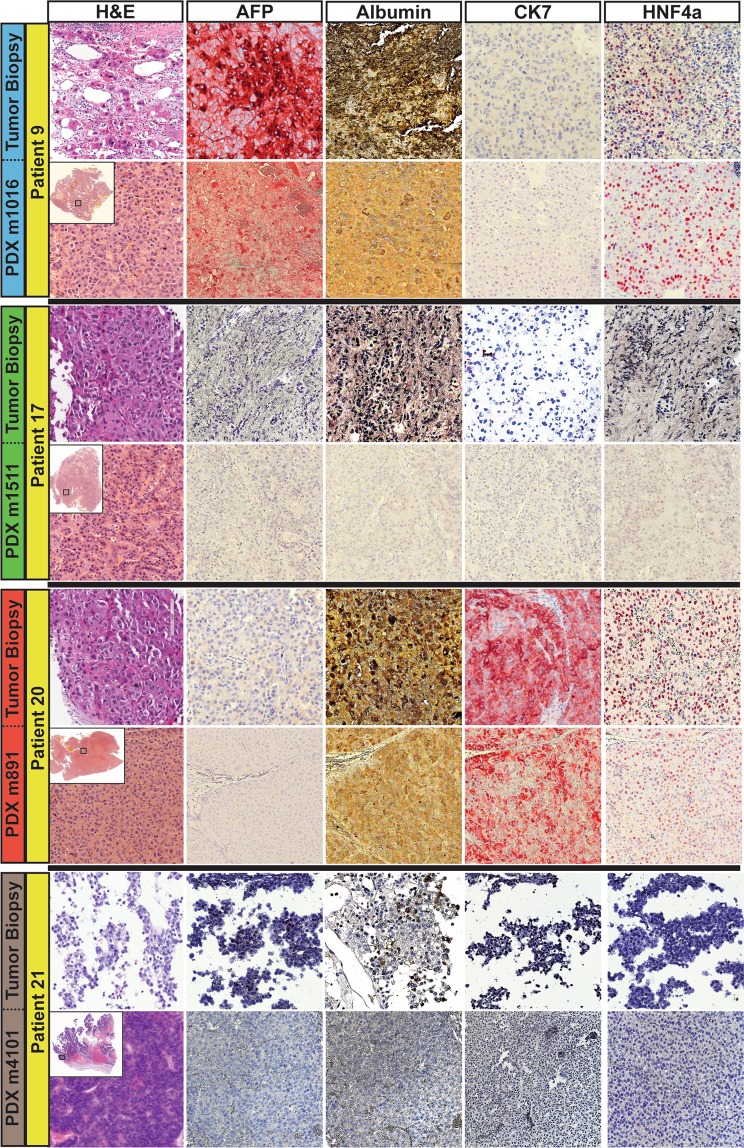
Molecular characterization of HCC-like xenografts generated from percutaneous biopsies implanted with Matrigel. Representative H&E sections (x200) of HCC/CHC tumor biopsies from 4 different patients (left-most panels) and the corresponding xenografts (right-most panels) demonstrate that xenografts resemble the morphology of the matching patient biopsies. Montages of each xenograft specimen are shown in the top left corner of each xenografts’ H&E together with a small black box indicating the region from which the representative images were taken. Immunohistochemical staining for HCC/CHC markers AFP, Albumin, CK7, and HNF4a demonstrates that despite molecular heterogeneity between the HCC/CHC xenografts from different patients, xenografts display similar patterns of expression to the matching patient biopsies.

We next examined the expression of HCC and hepatocyte markers in our CD45^−^ xenografts, each of which demonstrated features of HCC on H&E staining consistent with the matching patient biopsy. We found moderate to high intensity staining for albumin (hepatocyte marker) and cytokeratin-7 (CK7; cholangiocyte marker) in xenografts m891-m893, with weak to moderate expression of hepatocyte marker HNF4a (Fig. 3). This pattern of staining is consistent with a combined hepatocellular-cholangiocarcinoma (CHC) subtype^15^. Interestingly, the matching patient biopsy displayed similar patterns of marker expression and was diagnosed on clinical pathology as cholangiocarcinoma (Fig. 3, Supplementary Table 1). In further support of a CHC diagnosis, m891 was also shown to be AFP-, a well-established marker for HCC^16^ (Fig. 3). In contrast, analyses of xenograft m1016 showed strong expression of AFP, albumin, and HNF4a, with no expression of CK7, consistent with HCC, as was found in the matching patient biopsy (Fig. 3). Xenograft m1511 exhibited little-to-no expression of albumin, HNF4a, CK7 or AFP (Fig. 3). The matching patient biopsy, which was diagnosed on clinical pathology as moderately differentiated HCC with cirrhotomimetic growth, also showed no expression of AFP or CK7, with only rare cells expressing HNF4a or albumin. Lastly, xenografts m3585, and m4101-4103 were all negative for AFP, albumin, CK7, and HNF4a, with the exception of m4102 and m4103, in which 5–10% of cells showed weak-to-moderate expression of HNF4a. Interestingly, the matching patient biopsy was negative for all four markers, and was characterized by clinical pathology as poorly differentiated HCC with stem cell features. Taken together, these data suggest that minimally invasive percutaneous liver biopsies can be used with relatively high efficiency to generate patient derived xenografts of high fidelity to their cancer of origin for the two most common primary liver cancer subtypes. Moreover, this process may be augmented by the addition of Matrigel during the implantation process with a tumor take rate of 57% on a per patient basis.

## Discussion

To date, successful growth of HCC PDXs has primarily been achieved using surgical specimens from primary tumors. However, because only a minority of HCC patients are candidates for resection, surgical samples may inadequately reflect the full spectrum of tumor biology across the broader HCC population. Despite the small tissue volume obtained through percutaneous biopsies, we have shown that it is possible to generate HCC and CHC PDXs from intermediate and advanced stage HCC/CHC tumors using this technique. Given the growing role of biopsies in managing patients with HCC^17,18^, this capability holds important implications for the development of novel treatment paradigms in HCC that can be implemented in the context of current clinical workflows. Percutaneous biopsy-derived HCC PDX models could provide a platform to identify novel therapeutics as well as to predict patient response to existing therapies including sorafenib and regorafenib, two systemic therapeutics used to treat non-surgical candidates that have significant side-effects. The latter approach may reduce morbidity from side effects in patients for whom the drugs would not be effective. To this end, further studies are required to confirm that HCC PDX tumors developed through implantation of biopsy specimens in Matrigel display similar therapeutic responses as their tumor of origin. The development of techniques that maximize the success of PDX generation from percutaneous biopsy samples will be critical for the success of these strategies.

Given the small sample size, we cannot exclude confounding patient factors that may have affected the success of our implantations. While critical growth factors or extracellular matrix components in Matrigel seem to improve HCC/CHC cell engraftment, the mechanism through which these factors may inhibit the expansion of EBV-driven B-cells remains to be determined. In addition, the observation that when lymphoid tumors did form in the Matrigel cohort, they were observed at the site of implantation, rather than at distant metastatic sites as observed in the non-Matrigel cohort suggests a physical role for Matrigel in limiting the trafficking of lymphocytes into systemic circulation. These data emphasize the importance of continued vigilance for this phenomenon as well as the inclusion of other approaches to limit the formation of lymphoid tumors, such as treating mice with rituximab following implantation of biopsy samples^19,20^.

While the generalization of these findings should be made with caution, the inclusion of Matrigel in the implantation protocol coincided with significant improvement in the intended outcome. These data will serve to improve protocols for the generation of PDX models for individual HCC patients that are not operative candidates. Creating disease models with high fidelity to the original tumor tissue is of paramount importance for the successful development of individualized drug treatments and novel therapeutic approaches. The limited treatment options for patients with HCC, particularly those that are not candidates for resection, makes the generation of PDX models from biopsies a valuable approach to facilitate improved patient outcomes.

## Materials and Methods

### Patient tumor biopsies and engraftment in immunodeficient mice

As part of an ongoing observational clinical trial of subjects with HCC diagnosed on cross-sectional imaging using LIRADS criteria^14^, ultrasound guided percutaneous biopsies were obtained from patients using an 18 gauge BioPince core biopsy needle (Argon Medical, Frisco, TX). Three to five biopsies of the targeted lesion were acquired from each patient and used for histopathologic examination and xenograft generation. The investigation was approved by the Institutional Review Board (University of Pennsylvania Institutional Review Board #5 Protocol 823696) and informed consent was obtained from each patient. Haematoxylin and eosin (H&E) slides of a single biopsy from each patient were reviewed by a board-certified clinical pathologist with more than 25 years of experience (EEF).

Patient biopsies were engrafted into immunodeficient mice in strict accordance with the recommendations laid out in the Guide for the Care and Use of Laboratory Animals of the National Institutes of Health. The protocol was approved by the Institutional Animal Care and Use Committee at The University of Pennsylvania (Protocol Number: 803506). Individual biopsies were aspirated into a 1 mL syringe with 300ul of either PBS or 50% Matrigel (Corning, 354248) using an 18 G needle and injected subcutaneously into the right flank of 6–8-week-old male NSG mice (Jackson Labs strain #005557). Tumor growth was monitored weekly using a caliper and tumor volumes were calculated using the formula: V = 0.35 × (A x B)^(3/2), in which A and B are the long- and short-axis diameters of the tumor, respectively. Mice were euthanized and necropsies were performed when tumors reached a threshold long-axis diameter of 25 mm, or when mice showed signs of distress including weight loss ≥ 5 grams or failure to groom. Mice that did not show growth of subcutaneous tumors and did not meet euthanasia criteria based on weight loss or grooming were maintained in the mouse colony for at least 120 days. These mice were subsequently euthanized at 120–425 days following tumor implantation and were carefully examined for subcutaneous tumors and metastases at necropsy.

### Immunohistochemical staining and analysis

Standard immunohistochemistry (IHC) protocols were used to stain formalin-fixed, paraffin-embedded tissues (FFPE) or Optimal Cutting Temperature (OCT) compound-embedded tissues. Briefly, after deparaffinization (for FFPE samples) and rehydration of 4-µm-thick tissue sections, heat induced epitope retrieval was performed in 10 mM sodium citrate buffer (pH 6.0) using a pressure cooker for 20 min. For CK7 staining, protease based antigen retrieval (ACD Biosystems) was performed for 30 min at 37 °C. Endogenous peroxidase activity was then blocked with 3% hydrogen peroxide for 10 min at room temperature. The slides were then blocked for 30 min in StartingBlock T20. Primary antibodies diluted in 5% bovine serum albumin diluted in PBS with 0.2% Triton-X were incubated with sections overnight at 4 °C. Secondary antibodies of the appropriate species conjugated to either alkaline phosphatase or horseradish peroxidase (Vector Labs) were then applied for 30 min at room temperature. Color was developed using DAB (brown) or Vector Red substrate kits (Vector Labs) and sections were counterstained with Mayer’s hematoxylin. Primary antibodies utilized in this study included: rabbit anti-CD45 (Abcam, ab10558, 1:200), CD3/CD20 (cocktail, rabbit anti-human CD3 (clone SP7); mouse anti-human CD20 (clone L26), ThermoFisher, MV-2002-R7, neat), mouse anti-STEM121 (Clontech, Y40410, 1:1000), rabbit anti-AFP (Dako, A0008, 1:600), goat anti-albumin (Bethyl Laboratories, A80-229P, 1:200), mouse anti-CK7 (ThermoFisher, MA1-06316, 1:200), and mouse anti-HNF4a (Abcam, ab41898, 1:100). Histologic interpretations were confirmed by a hepatobiliary pathologist with over 25 years of experience (EEF).

### *In situ* hybridization (ISH) for EBV-encoded RNA (EBER1)

ISH for EBER1 was performed on FFPE sections using RNAscope 2.5 HD Brown Reagent Kit in combination with RNAscope Probe-V-EBER1 (Advanced Cell Diagnostics, Inc., Hayward, CA, USA) according to the manufacturer’s instructions.

### PCR for EBV-encoded RNA (EBER1)

EBER1 locus was amplified in explanted tumors using Eer1/F (5′-AAAACATGCGGACCACCAGC-3′) and Eer1/R (5′-AGGACCTACGCTGCCCTAGA-3′) primers^21^. Beta-Globin locus was amplified using PC04 (5′-CAACTTCATCCACGTTCACC-3′) and GH20 (5′-GAAGAGCCAAGGACAGGTAC-3′) primers^22^. PCR reaction contained 2.5ul 10x AmpliTaq Gold Buffer I, 1ul 10 mM dNTP mix, 1ul 5uM stock of both forward and reverse primers, 0.25ul AmpliTaq Gold polymerase and 63 ng of genomic DNA template in a final reaction volume of 25ul. Cycling conditions: 1. 10 min at 95 C, 2. 15 sec at 95 C, 3. 30 sec at annealing temperature, 4. 1 min at 72 C (35 cycles of steps 2–4), 5. 5 min at 72 C. Annealing temperatures: EBER1 PCR: 58.8 C, beta-globin PCR: 56.9 C.

### Ethics approval and consent to participate

Biopsy samples were acquired from study participants following informed consent and under an Institutional Review Board-approved protocol. Patient biopsies were engrafted into immunodeficient mice in accordance with institutionally approved protocols.

### Novelty and impact statement

More than 80% of patients with hepatocellular carcinoma (HCC) present with unresectable, incurable disease; however, the majority of existing models are derived from resection specimens limiting the availability of representative animal models. Herein, we describe our efforts to generate patient-derived xenografts (PDXs) from HCC biopsy samples to enable the generation of an animal model relevant to patients who are not candidates for curative therapies and are most in need of novel treatment options.

### Consent for publication

Informed consent for publication was obtained from each study participant.

## Supplementary information

Supplementary Table 1

## Data Availability

All data generated or analyzed during this study are included in this published article [and its supplementary information files].

## References

[1] Mak IW, Evaniew N, Ghert M (2014). Lost in translation: animal models and clinical trials in cancer treatment. Am. J. Transl. Res..

[2] Townsend EC (2016). The Public Repository of Xenografts Enables Discovery and Randomized Phase II-like Trials in Mice. Cancer Cell.

[3] Gillet J-P (2011). Redefining the relevance of established cancer cell lines to the study of mechanisms of clinical anti-cancer drug resistance. Proc. Natl. Acad. Sci. USA.

[4] Johnson JI (2001). Relationships between drug activity in NCI preclinical *in vitro* and *in vivo* models and early clinical trials. Br. J. Cancer.

[5] Gao H (2015). High-throughput screening using patient-derived tumor xenografts to predict clinical trial drug response. Nat. Med..

[6] Zhao Q (2016). Prognostic value of the expression of cancer stem cell-related markers CD133 and CD44 in hepatocellular carcinoma: From patients to patient-derived tumor xenograft models. Oncotarget.

[7] Zhang Y, Tang E-T, Du Z (2016). Detection of MET Gene Copy Number in Cancer Samples Using the Droplet Digital PCR Method. PLoS One.

[8] Ai J (2018). Preclinical Evaluation of SCC244 (Glumetinib), a Novel, Potent, and Highly Selective Inhibitor of c-Met in MET-dependent Cancer Models. Mol. Cancer Ther..

[9] Kissel M (2017). Antitumor effects of regorafenib and sorafenib in preclinical models of hepatocellular carcinoma. Oncotarget.

[10] Chen K, Ahmed S, Adeyi O, Dick JE, Ghanekar A (2012). Human Solid Tumor Xenografts in Immunodeficient Mice Are Vulnerable to Lymphomagenesis Associated with Epstein-Barr Virus. PLoS One.

[11] Timms JM (2003). Target cells of Epstein-Barr-virus (EBV)-positive post-transplant lymphoproliferative disease: similarities to EBV-positive Hodgkin’s lymphoma. Lancet.

[12] Thompson MP, Kurzrock R (2004). Epstein-Barr virus and cancer. Clin. Cancer Res..

[13] Allen UD, Preiksaitis JK (2013). Epstein-Barr Virus and Posttransplant Lymphoproliferative Disorder in Solid Organ Transplantation. Am. J. Transplant..

[14] Zhang L (2015). The extent of inflammatory infiltration in primary cancer tissues is associated with lymphomagenesis in immunodeficient mice. Sci. Rep..

[15] Broutier Laura, Mastrogiovanni Gianmarco, Verstegen Monique MA, Francies Hayley E, Gavarró Lena Morrill, Bradshaw Charles R, Allen George E, Arnes-Benito Robert, Sidorova Olga, Gaspersz Marcia P, Georgakopoulos Nikitas, Koo Bon-Kyoung, Dietmann Sabine, Davies Susan E, Praseedom Raaj K, Lieshout Ruby, IJzermans Jan N M, Wigmore Stephen J, Saeb-Parsy Kourosh, Garnett Mathew J, van der Laan Luc JW, Huch Meritxell (2017). Human primary liver cancer–derived organoid cultures for disease modeling and drug screening. Nature Medicine.

[16] Yamamoto K (2010). AFP, AFP-L3, DCP, and GP73 as markers for monitoring treatment response and recurrence and as surrogate markers of clinicopathological variables of HCC. J. Gastroenterol..

[17] Russo FP, Imondi A, Lynch EN, Farinati F (2018). When and how should we perform a biopsy for HCC in patients with liver cirrhosis in 2018? A review. Dig. Liver Dis..

[18] Finn RS (2016). HCC in Focus The Role of Liver Biopsy in Hepatocellular Carcinoma. Gastroenterol. Hepatol. (N. Y)..

[19] Corso S (2018). Rituximab Treatment Prevents Lymphoma Onset in Gastric Cancer Patient-Derived Xenografts. Neoplasia.

[20] Fujii E (2014). Characterization of EBV-related Lymphoproliferative Lesions Arising in Donor Lymphocytes of Transplanted Human Tumor Tissues in the NOG Mouse. Exp. Anim..

[21] Fujimuro M (2006). Multiplex PCR-based DNA array for simultaneous detection of three human herpesviruses, EVB, CMV and KSHV. Exp. Mol. Pathol..

[22] Qi ZL (2013). Comparison of three methods for the detection of Epstein-Barr virus in Hodgkin’s lymphoma in paraffin-embedded tissues. Mol. Med. Rep..

