# Supplementary material for: Establishment of hepatocellular carcinoma patient-derived xenografts from image-guided percutaneous biopsies
Source: Sci Rep. 2019 Jul 22;9:10546. doi: 10.1038/s41598-019-47104-9 (PMC6646301; doi:10.1038/s41598-019-47104-9)
Supplement: Supplementary file 1 — Supplementary Table 1 [file 41598_2019_47104_MOESM1_ESM.pdf]

Establishment of hepatocellular carcinoma patient-derived xenografts from image-guided percutaneous biopsies

David J. Tischfield MD, PhD[1,2,+], Daniel Ackerman PhD[1,2,+], Michael Noji[1,2], James X. Chen MD[1,2], Omar Johnson[1], Nicholas R. Perkons[1,3], Gregory J. Nadolski MD[1,2], Stephen J. Hunt MD, PhD[1,2], Michael C. Soulen MD[1,2], Emma E. Furth MD[4], Terence P. Gade MD, PhD[*,1,2]

1 Penn Image-Guided Interventions Laboratory, Perelman School of Medicine at the University of Pennsylvania, 3400 Spruce St., Philadelphia, PA 19104.

2 Department of Radiology, Perelman School of Medicine at the University of Pennsylvania, 3400 Spruce St., Philadelphia, PA 19104.

3 Department of Bioengineering, 210S 33rd St., Suite 240 Skirkanich Hall, Philadelphia, PA 19104

4 Department of Pathology and Laboratory Medicine, Perelman School of Medicine at the University of Pennsylvania, 3400 Spruce St., Philadelphia, PA 19104.

*Corresponding author
Terence P. Gade MD, PhD
Email: gadet@pennmedicine.upenn.eduOffice: (215) 573-9756
Fax: (215) 573-6725

Authorship note: [+]These authors contributed equally

Supplementary Info File:

Supplementary Table 1. Summary of clinical patient information and matching PDX data.

	PATIENT DATA										PDX DATA				
	Patient ID	Age (yr)	Sex	Ethnicity	Liver Disease	Pathology	IHC Marker Expression	LI-RADS Category	BCLC Stage	Prior TACE	PDX ID	Explanted Tumor Location	Pathology	IHC Marker Expression	Days to Euthanasia/Death
Biopsy Implantation Without Matrigel	9	45-50	M	White	None	Moderately Differentiated HCC	NA	5	C	Yes	m1001	NA	NA	NA	156
											m1002	NA	NA	NA	156
											m1003	NA	NA	NA	156
											m1004	NA	NA	NA	156
	12	65-70	M	White	HCV	Unavailable	NA	5	C	No	m1497	Splenic Tumors (x2)	Splenic Tumors: **Lymphoid** (H&E; IHC; ISH)	CD45(+), HNF4a(-), AFP(-), Albumin(-), CK7(-)	201
											m1498	Splenic Tumor (x1)<br>Hepatic Tumors (x8)	Splenic Tumor: **Lymphoid** (PCR)<br>Selected Hepatic Turmors: **Lymphoid** (PCR)	NA	173
											m1499	NA	NA	NA	201
											m1500	NA	NA	NA	201
	13	85-90	M	White	None	Poorly Differentiated HCC	NA	5	B	No	m1005	NA	NA		148
											m851	Lymph Node Tumor (x1)<br>Hepatic Tumor (x1)<br>Splenic Tumors (numerous)	Lymph Node Tumor: **Lymphoid** (H&E; IHC; ISH)<br>Hepatic Tumor: **Lymphoid** (PCR)<br>Splenic Tumors: **Lymphoid** (PCR)	Lymph Node Tumor: CD45(+), HNF4a(-) AFP(-), Albumin(-), CK7(-)	170
											m852	NA	NA	NA	204
	14	75-80	M	White	EtOH	Well Differentiated HCC	NA	5	C	No	m853	NA	NA	NA	140
											m854	NA	NA	NA	210
											m855	NA	NA	NA	210
Biopsy Implanatation With Matrigel	9	45-50	M	White	None	Moderately Differentiated HCC	HNF4a(+), AFP(+) Albumin(+), CK7(-)	5	C	Yes	m1016	Flank Tumor (x1)	Flank Tumor: **HCC** (H&E; IHC)	HNF4a(+), AFP (+), Albumin (+), CK7(-)	121
											m1027	Flank Tumor (x1)	Flank Tumor: **HCC** (H&E; IHC)	HNF4a(+), AFP (+), Albumin (+), CK7(-)	121
											m1029	NA	NA	NA	185
	15	55-60	M	White	None	Moderately Differentiated HCC with Clear Cell Features	NA	5	B	No	m1017	NA	NA	NA	262
											m1018	NA	NA	NA	262
											m1019	NA	NA	NA	262
											m1020	NA	NA	NA	262
	16	65-70	F	White	HCV	Moderately Differentiated HCC	NA	5	B	No	m1026	NA	NA	NA	425
											m1028	NA	NA	NA	425
											m1030	NA	NA	NA	425
	17	50-55	M	White	HCV	Moderately Differentiated HCC with Cirrhotomimetic Growth	HNF4a(+/--), AFP(-) Albumin(+/--), CK7(-)	5	B	No	m1511	Flank Tumor (x1)	Flank Tumor: **HCC** (H&E; IHC)	HNF4a(-), AFP(-), Albumin(-), CK7(-)	121
											m1512	NA	NA	NA	220
											m1513	NA	NA	NA	220
											m1514	NA	NA	NA	32
											m1515	NA	NA	NA	220
	18	55-60	M	White	HBV	Favor Cholangiocarcinoma; Cholangiohepatoma Not Excluded	NA	5	NA	No	m373	NA	NA	NA	286
											m374	NA	NA	NA	286
											m375	NA	NA	NA	286
	20	50-55	W	White	HCV	Cholangiocarcinoma	HNF4a(+/-), AFP(-) Albumin(+), CK7(+)	5	C	No	m891	Flank Tumor (x1)	Flank Tumor: **HCC** (H&E; IHC)	HNF4a(+/-), AFP(-), Albumin(+), CK7(+)	98
											m892	Flank Tumor (x1)	Flank Tumor: **HCC** (H&E; IHC)	HNF4a(+/-), AFP(-), Albumin(+), CK7(+)	139
											m893	Flank Tumor (x1)	Flank Tumor: **HCC** (H&E; IHC)	HNF4a(+/-), AFP(-), Albumin(+), CK7(+)	146
	21	60-65	M	White	HCV	Poorly Differentiated HCC with Stem Cell Features	HNF4a(-), AFP(-) Albumin(-), CK7(-)	5	B	No	m4687	Flank Tumor (x1)	Flank Tumor: **Lymphoid** (H&E; IHC; ISH)	CD45(+), HNF4a(-), AFP(-), Albumin(-), CK7(-)	163
											m4688	Flank Tumor (x1)	Flank Tumor: **Lymphoid** (H&E; IHC; ISH)	CD45(+), HNF4a(-), AFP(-), Albumin(-), CK7(-)	113
											m3585	Flank Tumor (x1)	Flank Tumor: **HCC** (H&E; IHC)	HNF4a(-), AFP(-), Albumin(-), CK7(-)	197
											m4101	Flank Tumor (x1)	Flank Tumor: **HCC** (H&E; IHC)	HNF4a(-), AFP(-), Albumin(-), CK7(-)	104
											m4102	Flank Tumor (x1)	Flank Tumor: **HCC** (H&E; IHC)	HNF4a(+/-), AFP(-), Albumin(-), CK7(-)	111
											m4103	Flank Tumor (x1)	Flank Tumor: **HCC** (H&E; IHC)	HNF4a(+/--), AFP(-), Albumin(-), CK7(-)	159
											m4104	Flank Tumor (x1)	Flank Tumor: **HCC** (H&E)	NA	180
											m4105	Flank Tumor (x1)	Flank Tumor: **HCC** (H&E)	NA	222

Legend: Lymphoid PDX HCC PDX

EtOH = Alcoholic
NASH = Nonalcoholic Steatohepatitis
HCV = Hepatitis C Virus
NA = Not Applicable
LI-RADS = Liver Imaging Reporting and Data System
"+" = strong expression
"-" = no expression
"-/+" = weak-to-moderate expression
"+/--" = generally negative but with small areas of weak, patchy expression
